# UV/Vis^+^ photochemistry database: Structure, content and applications

**DOI:** 10.1016/j.jqsrt.2020.107056

**Published:** 2020-09-01

**Authors:** Andreas Noelle, Ann Carine Vandaele, Javier Martin-Torres, Chenyi Yuan, Balabhadrapatruni N. Rajasekhar, Askar Fahr, Gerd K. Hartmann, David Lary, Yuan-Pern Lee, Paulo Limão-Vieira, Robert Locht, Kristopher McNeill, John J. Orlando, Farid Salama, Richard P. Wayne

**Affiliations:** a science-softCon, Auf der Burg 4, 63477 Maintal, Germany; b Planetary Aeronomy Division, BIRA-IASB, 3 av. Circulaire, B-1180 Brussels, Belgium; c Department of Computer Science, Electrical and Space Engineering, Luleå University of Technology, 97187 Luleå, Sweden; d Instituto Andaluz de Ciencias de la Tierra (CSIC-UGR), Av. de las Palmeras, 4, 18100 Armilla, Granada, Spain; e School of Geosciences, University of Aberdeen, Meston Building, King’s College, Aberdeen AB24 3UE, United Kingdom; f Oak Ridge Institute for Science and Education (ORISE), Office of Research and Development, U.S. Environmental Protection Agency, Athens, GA 30605, United States; g Atomic and Molecular Physics Division, Bhabha Atomic Research Centre, Mumbai 400 085, India; h American Chemical Society, Office of Research Grants, 1155 Sixteenth Street, NW, Washington D.C. 20036, United States; i William B. Hanson Center for Space Sciences, Department of Physics, University of Texas at Dallas, 800 West Campbell Road Richardson, TX 75080-3021, United States; j Department of Applied Chemistry and Center for Emergent Functional Matter Science, National Chiao Tung University, Hsinchu 30010, Taiwan; k Institute of Atomic and Molecular Sciences, Academia Sinica, Taipei 10617, Taiwan; l Atomic and Molecular Collisions Laboratory, CEFITEC, Department of Physics, Universidade NOVA de Lisboa, Campus de Caparica, 2829-516 Caparica, Portugal; m RU MolSys, Molecular Dynamics Laboratory, Department of Chemistry, Blg B6c, University of Liège, Sart-Tilman, B-4000 Liège 1, Belgium; n Institute for Biogeochemistry and Pollutant Dynamics, ETH Zurich, Universitätsstrasse 16, 8092 Zurich, Switzerland; o Atmospheric Chemistry Observations and Modeling Laboratory, UCAR, P.O. Box 3000, Boulder, CO 80307-3000, United States; p NASA-Ames Research Center, Space Science & Astrobiology Division, Mail Stop: 245-6, Moffett Field, CA 94035-1000, United States; q Chemistry Department, University of Oxford, South Parks Road, Oxford, OX1 3QR, United Kingdom

**Keywords:** Photochemistry, Spectroscopy, UV/Vis, Database, Radiative transfer

## Abstract

The “science-softCon UV/Vis^+^ Photochemistry Database” (www.photochemistry.org) is a large and comprehensive collection of EUV-VUV-UV–Vis-NIR spectral data and other photochemical information assembled from published peer-reviewed papers. The database contains photochemical data including absorption, fluorescence, photoelectron, and circular and linear dichroism spectra, as well as quantum yields and photolysis related data that are critically needed in many scientific disciplines.

This manuscript gives an outline regarding the structure and content of the “science-softCon UV/Vis^+^ Photochemistry Database”. The accurate and reliable molecular level information provided in this database is fundamental in nature and helps in proceeding further to understand photon, electron and ion induced chemistry of molecules of interest not only in spectroscopy, astrochemistry, astrophysics, Earth and planetary sciences, environmental chemistry, plasma physics, combustion chemistry but also in applied fields such as medical diagnostics, pharmaceutical sciences, biochemistry, agriculture, and catalysis. In order to illustrate this, we illustrate the use of the UV/Vis^+^ Photochemistry Database in four different fields of scientific endeavor.

## 1. Introduction

Photochemical data and information such as absorption spectra, fluorescence spectra, photoelectron spectra, circular and linear dichroism spectra, quantum yields etc. are important parameters needed in many scientific disciplines. Back in 1999 there was deemed to be a need for publicly accessible on-line databases containing such data and information in digital format (machine-readable). A first “UV/Vis Spectra of Atmospheric Constituents” CD-ROM [1] was published which contained at that time the largest collection of UV/Vis spectral data available free-of-charge. Based on this CD and the motivation to provide spectral data and information in digital format to the scientific community via the World Wide Web, the “UV/Vis Spectra Data Base” went on-line in August 2000 as a non-profit project. In the beginning, the on-line database contained about 1200 spectra/datasheets for 120 substances and the compiled data extended beyond atmospheric research to allow for interdisciplinary application. Although other databases existed at that time, they were mainly focused on infrared studies like HITRAN [2, 3] or GEISA [4, 5] or were focused on providing UV cross sections of gaseous molecules of atmospheric interest [6].

To enable platform independent usability, both the spectral data as well as the datasheets (meta-data such as publication, authors, source, wavelength range, temperature, pressure, phase etc.), are available as plain ASCII text. To guarantee the high quality standard of the fast growing “UV/Vis^+^ Spectra Data Base”, an international “Scientific Advisory Group” (SAG) was established in 2004, and the database was operated in accordance with the “Open Access” definitions and regulations of the CSPR Assessment Panel on Scientific Data and Information (International Council for Science, 2004, ICSU Report of the Committee on Scientific Planning and Review Assessment Panel on Data and Information [7]). Since 2004, in addition to publishing the on-line database every 2 years, a mirror of the on-line database has been published on CD-ROM. The latest edition in the “ science-softCon UV/Vis^+^ Spectra Data Base” series was published in 2019 [8]. The on-line database currently (as of January 2020) contains about 14,000 spectra/datasheets as well as 5200 graphical representations for about 3000 substances and is subdivided into 28 substance groups (e.g. hydrocarbons, pharmaceuticals, pesticides, polycyclic aromatic hydrocarbons, etc.). The database is updated weekly. In addition to the inclusion of new data, a main focus of the database is the preservation of data from older publications.

As mentioned by the CSPR Assessment Panel on Scientific Data and Information, database maintenance and management are costly [7]. Collection of data, preparation of metadata, and provision of professional data management expertise and institutional support for data dissemination and permanent archiving will add to the overall expense of specific research projects and maintaining the larger research infrastructure.

“Full and open access” to data implies equitable, non-discriminatory access to all data that are of value for science. It does not necessarily equate ‘free of cost’ at the point of delivery.

There are several economic models for providing scientists with access to data for research and education [7]. The “UV/Vis^+^ Photochemistry Database” allows free and open access to all meta-data, and cost-recovery pricing for data (or data licenses) in order to support the full data infrastructure. Different charged subscriptions giving full-access to the data are available: for example a yearly campus-wide license provides full access to all data and information for less than 1 USD per day (for universities, governmental organizations, non-profit organizations) and a “One-time registration” license allows the subscriber to indefinitely have access to all data and information. Both licenses includes a copy of the 12th edition of the “UV/Vis^+^ Spectra Data Base” CD-ROM [8]. In addition, those colleagues who support us in maintaining the database through the provision of new or missing data and information can get personal free-of-charge access to all data and information. More information is available at www.photochemistry.org.

## 2. Database structure and content

The database contains spectral information (gas, liquid and solid phase) from extreme ultraviolet to near infrared spectral region (EUV-VUV-UV-Vis-NIR) and related data (e.g. information concerning publications on quantum yield studies or photolysis studies) from published peer-reviewed papers. Besides absorption spectra, which comprise most of the available data, fluorescence spectra, photoelectron spectra, circular and linear dichroism spectra, quantum yields etc. are available. The database is structured into 28 categories which outlines only a rough classification.

The data sheets provide meta-data (substance name, formula and CAS number, data source, full reference, including title, authors, journal and DOI when available, spectral range and resolution, temperature, pressure, phase, etc.), as well as data in various forms obtained and presented in the literature. This includes, for example, absorption data measured over a specific wavelength/energy range in tabulated form. In many applications (e.g. quantum yield studies or photolysis studies), the absorption cross section (*σ*) or the molar extinction coefficient (*ε*) at a specific wavelength (*λ*) is determined, and these single wavelength data are also included in the database. For many substances temperature dependent data are available.

**Fig. 1.**
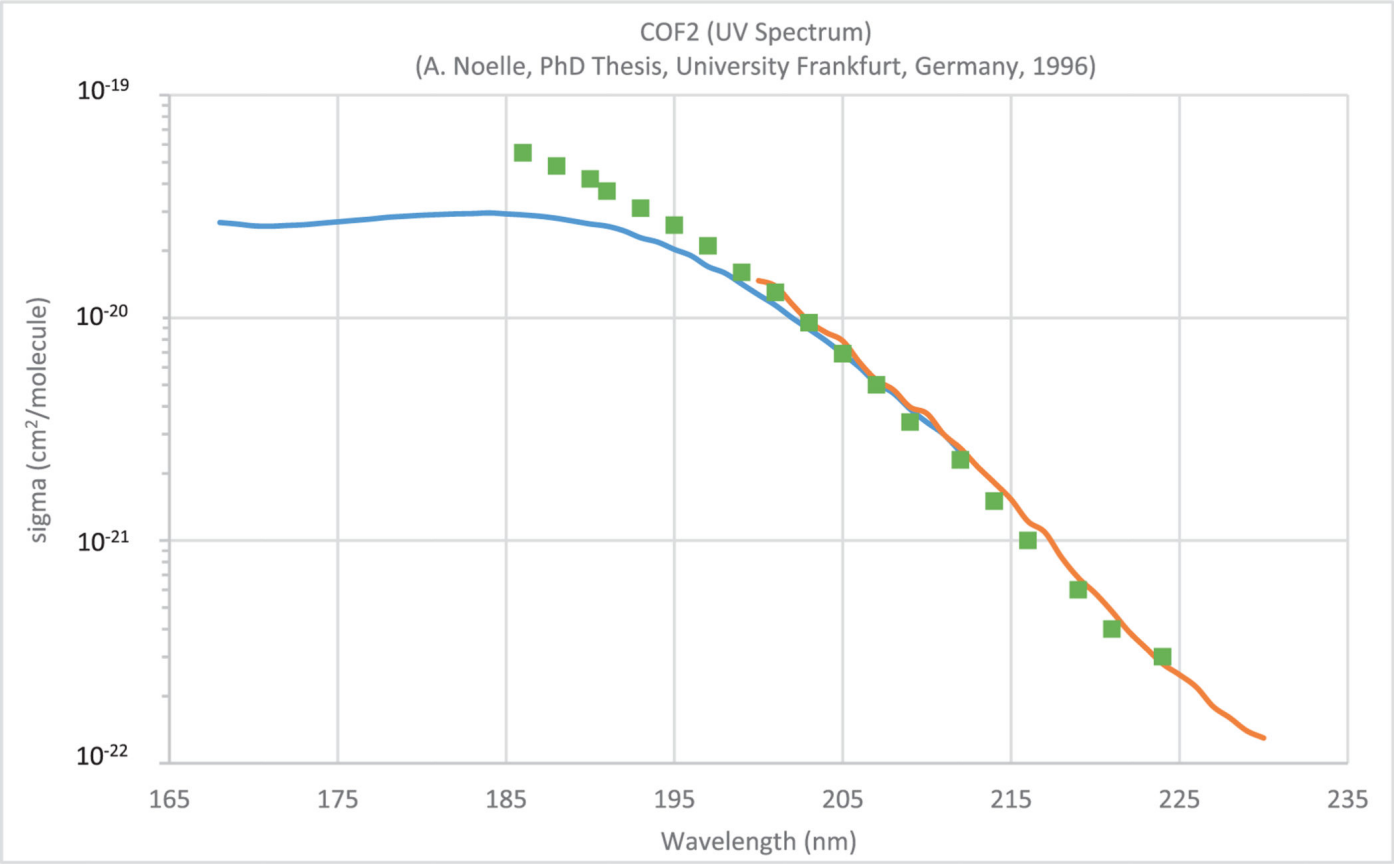
UV absorption spectrum of COF_2_ obtained by Noelle [9] (blue curve), Noelle et al. [10] (red curve), and Molina and Molina [11] (green squares).

Most of the available data are from published peer-reviewed papers (> 98%), data presented at scientific meetings and conferences are also available (< 1%), as well as data from PhD theses, reports and unpublished material (< 1%). As an example of the database structure and contents, absorption data of carbonyl fluoride (COF_2_) from three different sources are presented in Fig. 1. The data sets are as provided by the authors or listed in the relevant publications. To enable a platform independent usability, all data are provided as plain ASCII text.

During more recent years, almost 3000 graphical representations, mostly from older publications have been digitized and added to the database. In addition, we have converted more than 100 datasets from “floppy discs” and hence prevented these data from being lost as technology has evolved.

**Fig. 2.**
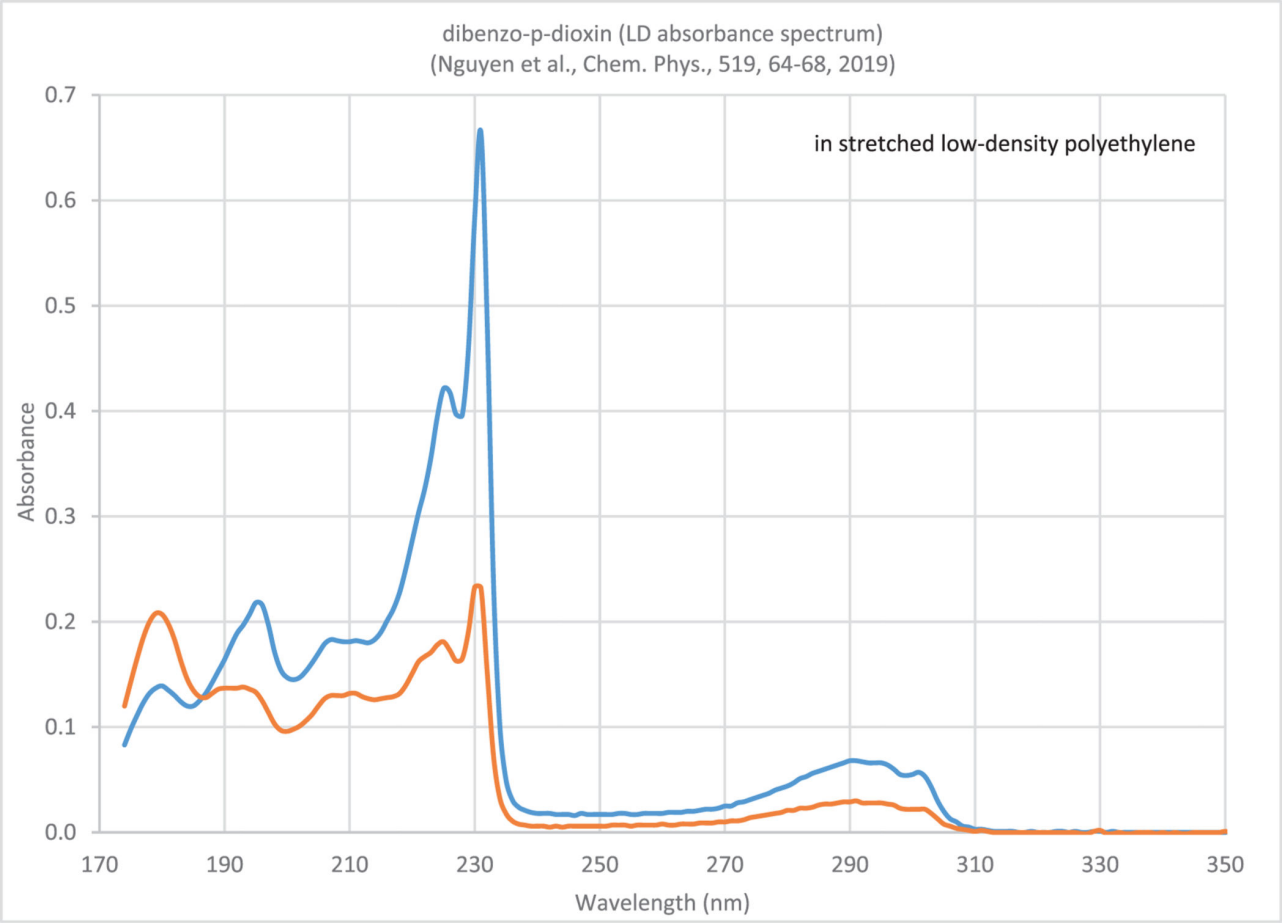
Linear dichroism absorbance spectrum of dibenzo-p-dioxin [12]. The absorbance curves were recorded with the electric vector of the sample beam parallel (blue line) and perpendicular (orange) to the stretching direction of the polyethylene polymer.

Since 2019 the database has been extended to include circular and linear dichroism spectral data (Fig. 2). The absorbance curves were recorded with the electric vector of the sample beam parallel and perpendicular to the stretching direction of the polyethylene polymer.

In addition to the data relevant to the different species, the following data and information (including software) are available to all interested scientists:

### AutoChem

AutoChem is an automatic computer code generator and documentor for chemically reactive systems written and updated by David Lary of NASA Goddard Space Flight Center since 1993. It was designed primarily for modeling atmospheric chemistry, and in particular, for chemical data assimilation (see [13] and references herein).

### Daily Solar Irradiances

The solar irradiance data was kindly provided by Judith Lean of the Naval Research Laboratory (NRL). Daily files are available from 1975 to 2004. The solar spectra in 203 bands from 0.121 – 0.859 *μ*m are given.

### MAS

(Millimeterwave Atmospheric Sounder), provided by G.K. Hartmann Max Planck Institute (MPI) for Solar System Research (formerly MPAe – Max Planck Institüt für Aeronomie) [14].

MAS was part of NASA’s Atmospheric Laboratory for Applications and Science (ATLAS) Spacelab shuttle mission and provides a database for the study of changes in the middle atmosphere. MAS is a remote sensing instrument for passive sounding of the Earth’s atmosphere, designed to study the formation and destruction of ozone by measuring emission lines of ozone, water vapor, and chlorine monoxide from the space shuttle. The millimeter-wave radiation emitted by the atmosphere in the altitude range between 10 km and 100 km has been measured at 61, 62, 63, 183, 184 and 204 GHz.

### SUMER

(Solar Ultraviolet Measurements of Emitted Radiation) provided by MPI for Solar System Research (W. Curdt) [15, 16].

SUMER was a UV telescope and spectrometer designed for high-resolution observations of the Solar atmosphere in the extreme ultraviolet wavelength range from 50 to 160 nm. The instrument was part of the payload on-board the ESA/NASA spacecraft SOHO.

## 3. Database applications

The interdisciplinary usability of the “UV/Vis^+^ Photochemistry Database” is shown on the basis of four different applications.

### 3.1. Modeling direct phototransformation of aquatic organic contaminants under environmentally relevant conditions

An increasing number of synthetic organic compounds (SOCs) are being used and discharged to the aquatic environment. Under the influence of natural sunlight, some of these SOCs are likely to transform through photolysis to more toxic products or to be photo-persistent. To meet the large data demand for risk assessment of SOCs, a cheminformatics-based direct photolysis reaction library is being developed as part of the United States Environmental Protection Agency’s Chemical Transformation Simulator (CTS) project to predict the direct photolytic transformation products formed from organic contaminants in waters [48]. Both the literature and data service of UV/vis^+^ Photochemistry Database are useful in developing and applying such a predictive tool.

Central to the direct photolysis predictive tool is a large compilation of relevant literature about the direct photo-transformation pathways of SOCs. The science-softCon UV/Vis^+^ Photochemistry Database’s literature service has served as a convenient and reliable literature source. Up to Janurary 2020, 109 of the 390 compounds compiled by CTS’s direct photolysis reaction library are logged in the UV/Vis^+^ Photochemistry Database. Compared with the larger Reaxys database, which also has UV–Vis spectrum and photolysis information, the UV/Vis^+^ Photochemistry Database is of no or low cost, focused on photochemistry, easier to locate relevant information, and, most importantly, has digitalized spectra for analysis.

When applying the direct photolysis predictive tool, users will be able to predict transformation products. However, direct photolysis rate information is also important to know whether a contaminant is degraded and whether its products can be formed at an environmentally relevant rate. The UV–vis spectrum and quantum yield logged in the science-softCon UV/Vis^+^ Photochemistry Database can be used for such a rate estimation according to the following equation [17, 18]:

(1) $$k\approx 2.303\int I_{\lambda }\varepsilon_{\lambda }\varphi_{\lambda }d\lambda \approx 2.303\varphi \text{Σ}I_{\lambda }\varepsilon_{\lambda }\text{Δ}\lambda$$

Where *k* is the first-order rate constant for the direct photolysis of the contaminant in pure water, *I*_λ_ is the solar irradiance at a certain wavelength λ, *Ɛ*_λ_ is the molar extinction coefficient of the contaminant at wavelength λ, and *φ*_λ_ is the quantum yield of the photodegradation process at wavelength λ. Δλ is often 1 nm if we use *I*_λ_ and *Ɛ*_λ_ at every relevant wavelength. The first “≈” in the equation assumes that the solution is optically thin (absorbance < 0.02) and ignores the effect of reflection on pathlength, and the second “≈” assumes that *φ* is the same across the corresponding wavelengths and uses finite Δλ for integration.

**Fig. 3.**
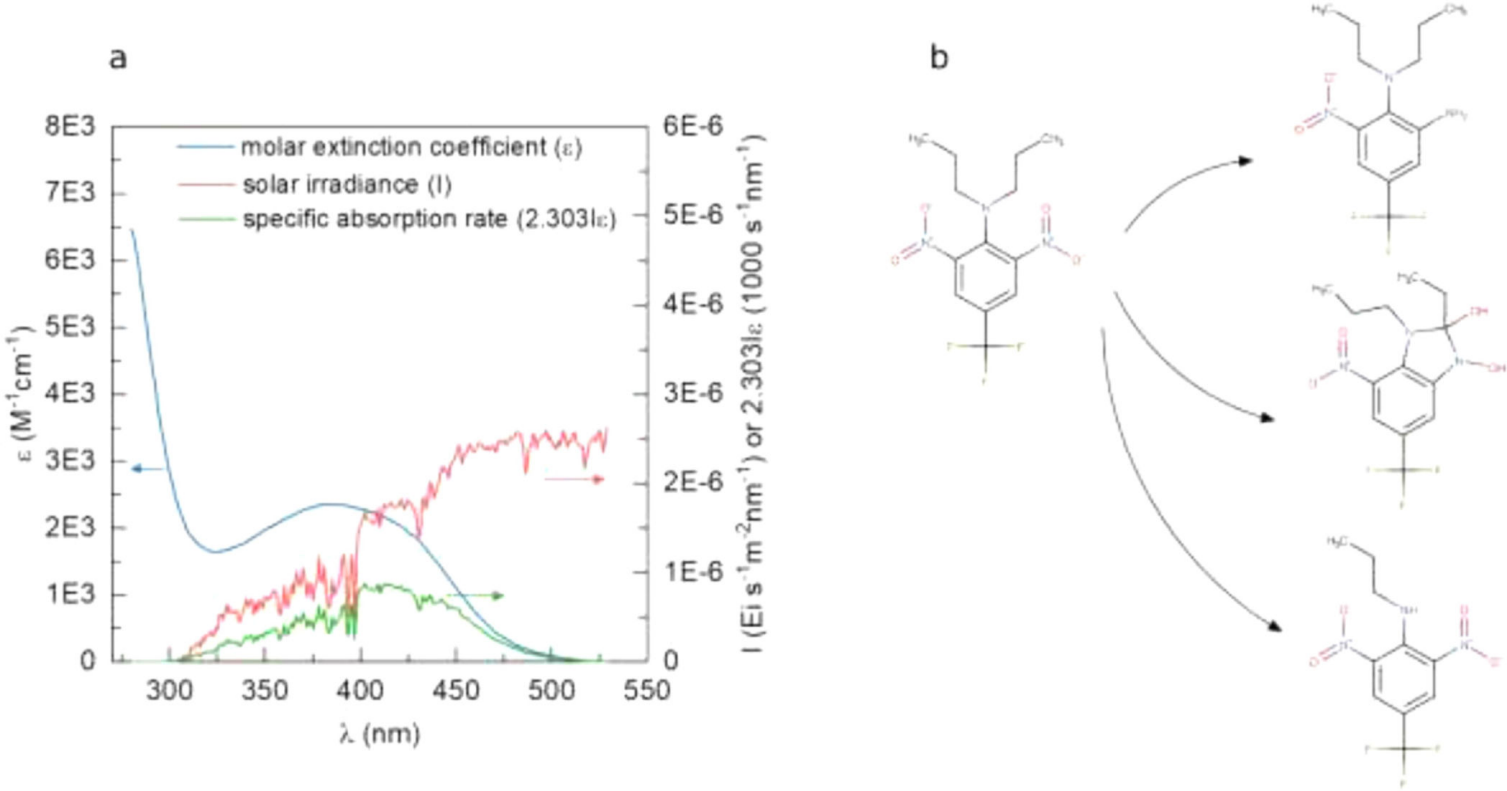
Degradation rate estimation (a) and product prediction (b) for direct photolysis of trifluralin.

Table 1 shows an example back-of-the-envelope estimation of the direct photolysis rate at water surface of a strongly-absorbing pesticide, trifluralin, a moderately-absorbing explosive compound, RDX (1,3,5-trinitroperhydro-1,3,5-triazine), and a weakly-absorbing pesticide, metolachlor using the information obtained from the science-softCon UV/Vis^+^ Photochemistry Database and a reference solar spectrum of a mid-latitude mid-summer day[18]. The estimation was conducted manually but can also be performed using software such as GCSOLAR [19] and APEX [20]. An example of the detailed spectra is plotted in Fig. 3.a for trifluralin. All three compounds were predicted to photodegrade to products: three 1st-generation products for trifluralin as shown in Fig. 3.b, one for RDX, and three for metolachlor. However, the estimated half-lives suggest that metolachlor is less photolabile compared to trifluralin. Therefore, products of metolachlor are less likely to be photochemically formed in the environment compared to that of trifluralin, providing the ratios of product formation over parent degradation are similar and the photo-lability of 1st-generation products are similar. As illustrated in this example, the science-softCon UV/Vis^+^ Photochemistry Database provides a literature-based method of estimating direct photolysis rates, adding another piece of information in identifying photolabile contaminants and their product formation. A larger amount of more accurate data, especially in near-UV to visible range and in aquatic phase, would lower the current large uncertainty between rate estimation and experimental data (Table 1 for metolachlor) and eventually allow automation of such calculations.

### 3.2. Application of UV VUV absorption spectra for air quality, photochemistry and climate on Earth

**Fig. 4.**
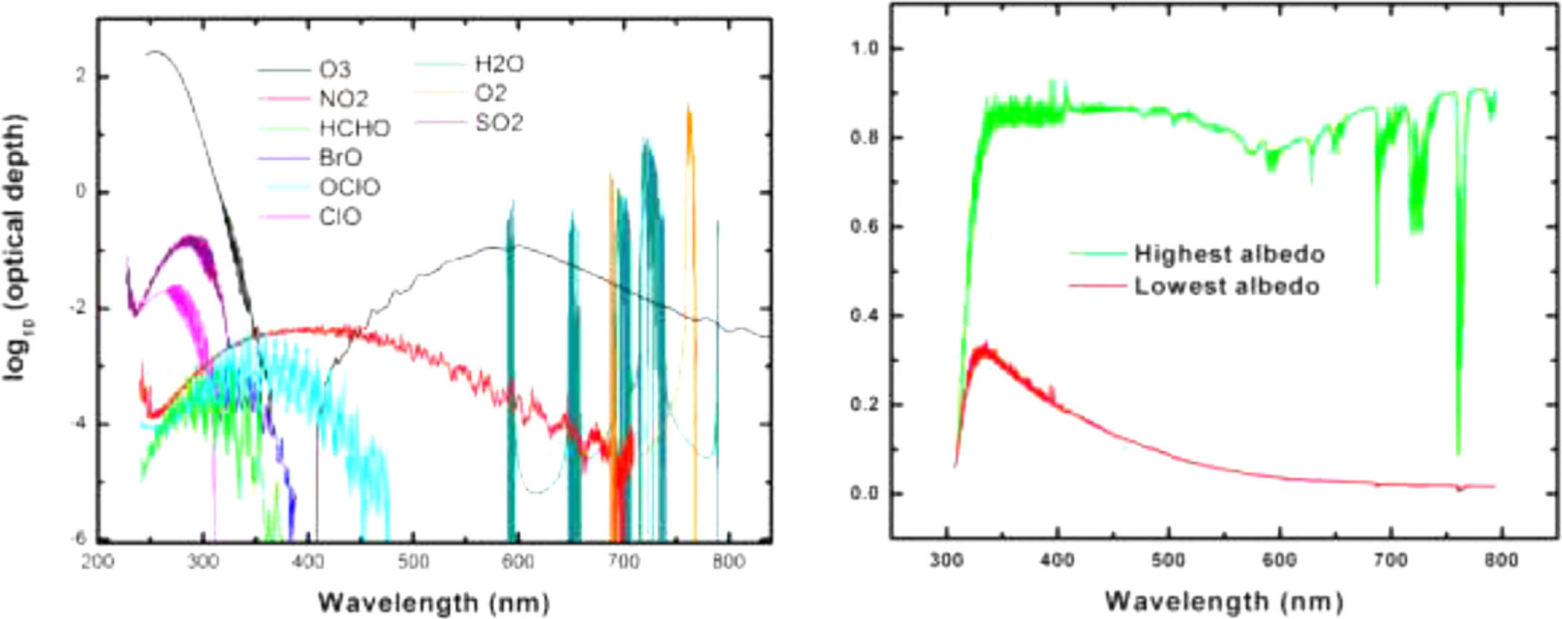
Left: Absorptions due to molecules that are now commonly measured from space in the nadir geometry; Right: Back scattered albedo spectra from GOME measurements for two extreme examples. The highest albedo scene, corresponding to full coverage by high clouds, is white and quite bright, due to the cloud reflectance; the lowest albedo case is a cloud-free scene over the ocean [30].

**Fig. 5.**
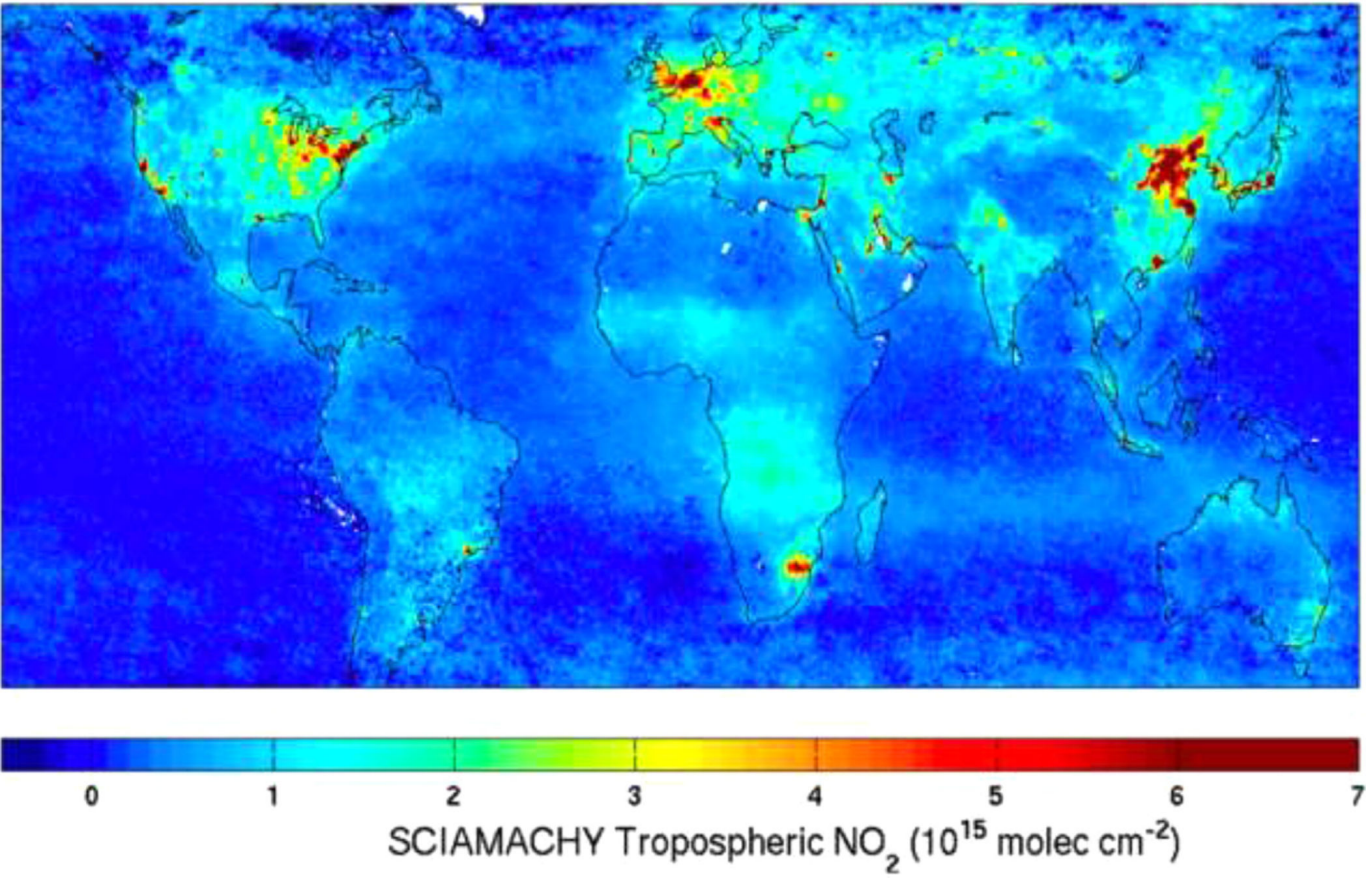
Tropospheric NO_2_ retrieved from SCIAMACHY measurements in the 425–450 nm spectral region. Pollution on urban scales is readily measured globally [30].

The UV–VIS spectral region is of fundamental importance for understanding air quality, the photochemistry of the troposphere and stratosphere, and cloud coverage, and their implications for climate change. In particular, by using the unique absorption signatures of gas species (see Fig. 4), abundances of a range of air pollutants like ozone (O_3_), nitrogen dioxide (NO_2_), formaldehyde (HCHO), and glyoxal (CHOCHO) (see Fig. 5) can be inferred from satellite UV–Vis spectroscopy measurements, as well as, for example, species involved in the destruction of polar ozone (BrO, IO and OClO).

Satellites’ observations can be used together with climate models to understand the relationship between the atmospheric composition and climate. The combination of UV/Vis radiation allows the retrieval of atmospheric parameters that are key in the research of climate, air quality and polar chemistry:

- Relevant chemical species in the troposphere;
- Spectral optical density of aerosols in a full spectral range;
- Cloud cover;
- Cloud height.

The continuous improvement of data provided by the “science-softCon UV/Vis^+^ Photochemistry Database” is fundamental in achieving the required accuracy of the ever more demanding spectral measurements of the atmosphere.

### 3.3. Application of UV and VUV absorption spectra for breath gas analysis

**Fig. 6.**
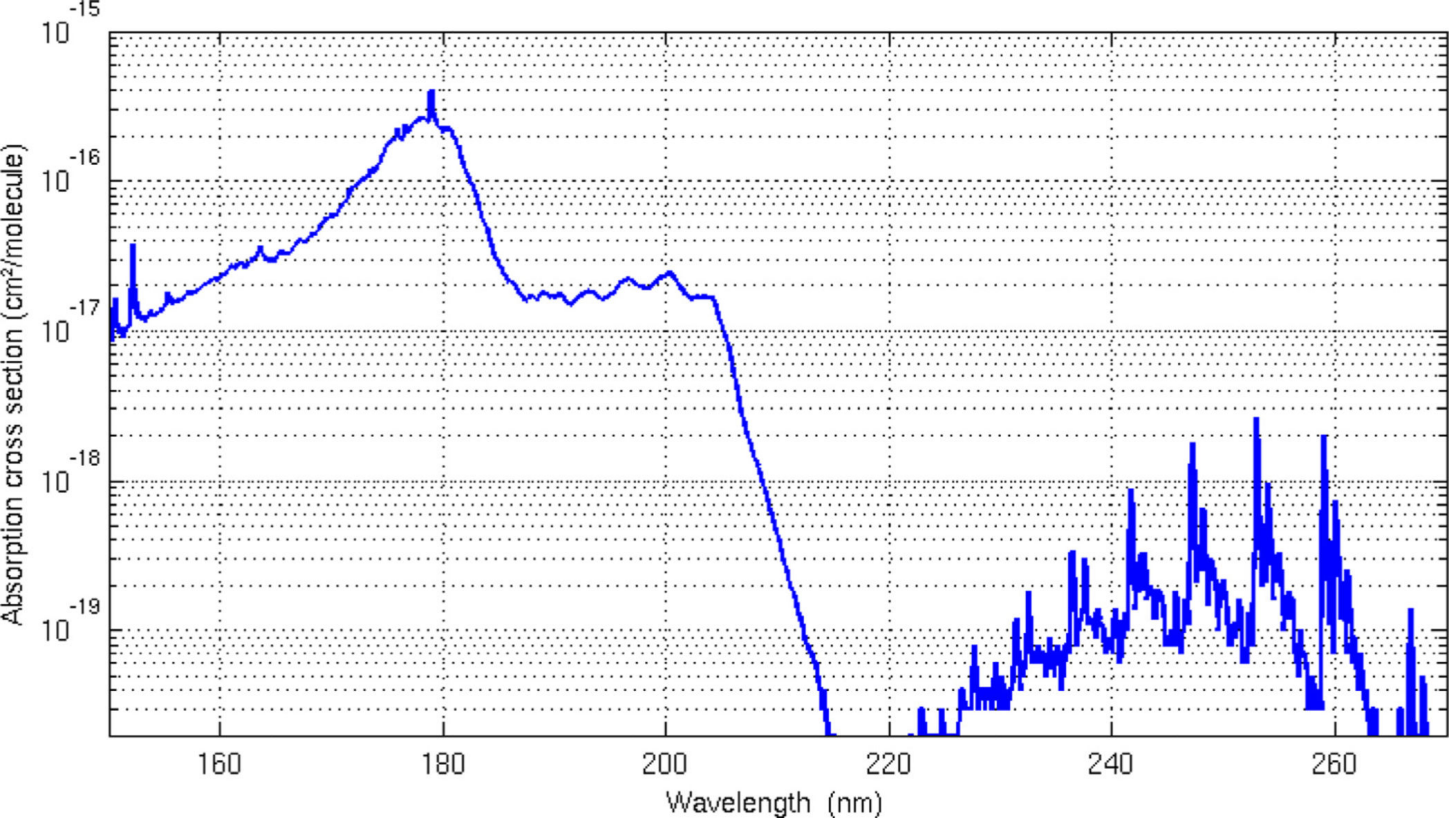
Absorption cross-section of benzene [40]: in the VUV region the cross-section values are between 2 and 3 orders of magnitude larger than in the UV range.

The study of Secondary Organic Aerosols (SOAs) which are the reactive products of gas-phase photo-oxidation of both naturally-occurring and man-made volatile organic compounds (VOCs) can be studied in the UV-VUV spectral region. Volatile organic compounds are also released by animal and human organisms with normal metabolic activity or due to pathological disorders because of diseases, infections and/or internal injuries. Breath gas analysis, which works on the principles of understanding changed level of concentrations of VOCs in exhaled breath from human beings or animals, can indicate the onset or progression of a disease [31]. For example in human beings, the presence of acetone in the breath can be observed in the case of untreated or poorly treated diabetes [32], and pentanes and carbon disulfide have been associated in case of schizophrenia [33] etc. However there are many short-comings with existing gas detection techniques such as gas chromatography, mass spectroscopy, and infrared spectroscopy which include complications such as sample pre-concentration, imperfect existing profile recognition, time-consuming, nearly impossible real-time measurements, limited characteristic VOC identification and detectable components, and definite differentiation of isomeric and isobaric molecules [34]. These limitations are compounded by complications as VOCs in breath gas samples are governed by a combination of their concentration in blood, the retention time of the compounds in the lung and airway tissue (inhalation – exhalation cycle), and last but not least, the VOC blood/air partition coefficient [31]. To overcome these limitations for using breath gas analysis as a diagnostic tool, a new concept of using GC–MS technology in conjunction with ultraviolet absorption spectroscopy is being evaluated by developing laboratory equipment [35–37]. In particular the use of the vacuum ultraviolet (VUV) region improves the detection of such compounds, because absorption cross sections in this spectral range are usually larger by orders of magnitude than in the UV or in the IR. This is illustrated in Fig. 6, where the VUV-UV cross-section of benzene is shown. The higher sensitivity in the VUV range allows fast and sensitive spectroscopic detection. Moreover it is also possible to derive structural and isomer-related information from the VUV spectra. Especially small oxygenated substances like short chain fatty acids, small n-alkanols (C2–C4), esters, aldehydes and ketones, which are abundantly present in breath gas, show good VUV sensitivity with rich structural information [38]. Isoprene has been reported as a candidate for monitoring cholesterol metabolism and has been successfully measured in breath [39].

The existing ultraviolet absorption spectra and accurate spectroscopic information of molecules available from “science-softCon UV/Vis^+^ Photochemistry Database” play a major role in identifying their presence and finger printing them in a specific isomeric or isobaric phase as a good starting point. Understanding the presence of different molecules and their concentrations in exhalation and inhalation cycles in human beings and animals as a consequence of biochemical changes is an important driver for developing analytical methods such as breath gas analysis with UV-VUV spectroscopy as an analytical tool for medical diagnostics. Spectroscopic investigations of biologically important molecules cataloged in the present database and their spectral responses in mixtures with atmospheric gasses provide important inputs in understanding their role in physiological processes. These inputs in turn generate important data required for understanding the presence of disease, disease management and usefulness of new drugs under trial for a specific disease.

### 3.4. Remote sensing and modeling of Mars and Venus atmospheres

**Fig. 7.**
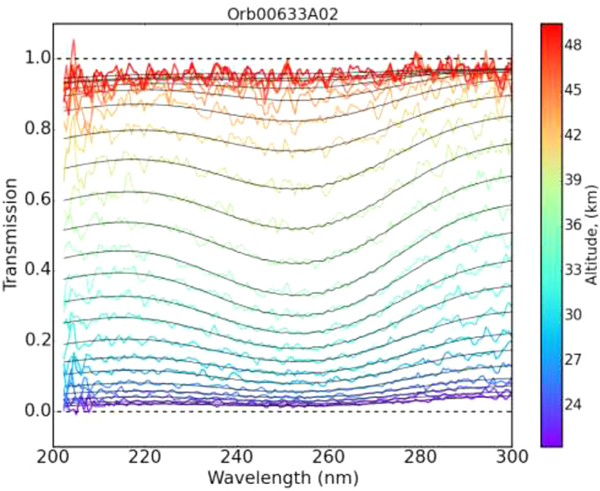
Example of SPICAM transmittance spectra obtained during one solar occultation observation. The color indicates to altitude probed from low altitudes (blue) to high altitudes (red). Black lines are fitted spectra using (Eq. (2)) [Figure from [42]].

Today more and more complex instruments are used for remote sensing of the atmosphere of the Earth and other planets of our Solar System or even further, with the development of missions and instrumentation, not only to search for exoplanets, but now to deliver spectroscopic survey of their atmospheres. In particular, instruments probing the UV-visible spectral range have been used to characterize the atmospheric composition of a wide variety of planets. For example, the SPICAM (SPectroscopie pour l’Investigation des Caractéristiques Atmosphériques de Mars) instrument on board the ESA’s mission Mars Express, is a remote sensing spectrometer observing Mars in the ultraviolet and the near infrared [41]. The UV range covers the absorption due to CO _2_ and O _3_ (118–320 nm). The instrument probes the Martian atmosphere in nadir geometry (looking down to the surface of the planet) or through stellar and solar occultation. In solar occultation (i.e. looking at the Sun through the atmosphere), the signal received by the instrument is given by:

(2) $$I(\lambda )=I_{Sun}(\lambda )e^{-\tau \left(\lambda ,0,S_{obs}\right)}$$

where *I*_*Sun*_ represents the light intensity of the source, here the Sun, placed at the starting point of the raypath situated at the distance *s*_*obs*_ from the observer, and τ is the optical depth along the line of sight, generally expressed as

(3) $$\tau \left(\lambda ,s_{1},s_{2}\right)=\int_{s1}^{s2}\sigma_{a}(\lambda ,P,T)\text{n}(s)ds$$

where *σ*_*a*_ is the absorption cross-section and *n* is the density of the species. The integration is done along the line of sight, i.e. through the atmosphere, considering the temperature (T) dependence of the cross section, as temperature varies substantially from the lower layers to the upper layers. This spectroscopic investigation is based on the use of absorption cross-sections measured in the lab and inventoried in databases such as the Spectra Database described in this work. In the case of SPICAM, we considered the temperature dependence of the CO_2_ and O_3_ absorption cross sections [42]. Fig. 7 illustrates a typical analysis of a set of spectra obtained during one solar occultation observation, in which the signature of ozone is quite easily observed. A similar approach was considered for the analysis of the UVIS channel of the NOMAD instrument on board ExoMars TGO [43].

However, the accurate knowledge of cross-sections is not only required for the direct analysis of the observations, but is also crucial for the modeling of such atmospheres, through 1D photochemical to 3D Global Circulation models. The continuity equation

(4) $$\frac{\partial n}{\partial t}=P-nL-\frac{\partial \text{Φ}}{\partial z}$$

governs the chemical composition within an atmosphere, where *z* is the altitude, *n* is the number density of the species, *P* and *L* are its production and loss rates, and Ф is its upward transport flux. The production and loss rates are provided from all chemical and photochemical reactions that produce or consume the species. In particular, the photodissociation rate (J) is given by:

(5) $$J(z)=\frac{1}{2}\int \varphi (\lambda ,T(z))\sigma_{a}(\lambda ,T(z))F(\lambda ,z)d\lambda$$

where *σ*_*a*_ is the absorption cross-section, *φ* is the quantum yield of the photodissociation process and *F* is the actinic flux at the altitude *z* where temperature *T* prevails. But absorption, and therefore the effect of the cross-section, is also hidden within *F* (λ,z) which corresponds to the radiation flux reaching the altitude z and is given by

(6) $$F(\lambda ,z)=F_{0}(\lambda )e^{-\tau (\lambda ,z)/\mu }$$

if no scattering is considered within the atmosphere. *F*_*0*_ is the radiation flux at the top of the atmosphere, *μ*= cos(*θ*) with *θ* the solar zenith angle and *τ*(λ,z) is the optical depth which includes the absorption by all gasses, Rayleigh scattering and aerosols extinction from the top of the atmosphere down to the altitude *z*.

Available cross-sections are usually derived from ambient temperature measurements, some are provided at low temperatures corresponding to atmospheric conditions encountered on Earth. However, data at temperatures and pressures found on other bodies, in particular at high temperatures and low pressures, are very scarce and often restricted to small spectral ranges. This lack of data has a direct impact both on the retrieval of abundances using UV spectra (Equ.3), but also on the simulation of the photochemical processes within these atmospheres. In particular CO_2_ is an important species which has a large impact on the photochemistry of atmospheres of Mars and Venus, controlling the transmissivity of the atmosphere and therefore the photolysis rates of the other species. The impact of the temperature dependence of CO_2_ cross-section has been analyzed in several recent papers [44–46], showing the importance of considering the temperature dependence on a wide range of temperature as recently imported into the database [46, 47]. Indeed, the absorption of the UV flux increases substantially together with temperature since CO_2_ absorption cross sections are larger at higher temperatures. Using room temperature reference data to model CO_2_-rich atmospheres, the absorption of the UV flux and therefore the photochemical rates are underestimated.

## 4. Outlook

The “science-softCon UV/Vis^+^ Photochemistry Database” is continually evolving and growing. As of January 2020, it includes about 14,000 spectra/datasheets as well as 5200 graphical representations for about 3000 substances and is subdivided into 28 substance groups (e.g. hydrocarbons, pharmaceuticals, pesticides, polycyclic aromatic hydrocarbons etc.) This is a tremendous effort and requires a lot of manpower, not to mention technical infrastructure. We hope that the database proves useful to the scientific community and will facilitate their day-to-day work.

Since the support by the scientific community is crucial for such a photochemistry database, we would like to encourage all colleagues to assist us in maintaining the database and join our initiative “Photochemical Data and Information Sharing Platform - Share Photochemical Data & Information, Find Answers”.

This initiative should develop the photochemical database towards a photochemical data sharing platform. The advantage of such a photochemical data sharing platform is that the more scientists provide their data for inclusion into the database the better is the chance for all users to find specific photochemical data within the database. In addition, the platform becomes increasingly beneficial for use across multiple disciplines.

## Figures and Tables

**Table 1:** Estimates of direct photolysis rates in mid-latitude mid-summer sunlight.

compound	$2.303{\text{ΣI}}_{\lambda }\varepsilon_{\lambda }\text{Δ}\lambda \left({\text{s}}^{-1}\right)$^a^	matrix for UV-vis spectrum	ϕ^b^	half-life		
				calculated if *ϕ* = 1	calculated using selected *ϕ*	natural sunlight experiment^c^
trifluralin	8.1 × 10^−02^	Methanol [21]	1.4 × 10^−3^ at 310–410 nm [22], 6 × 10^−1^ at 254 nm [23]	9 s	1.7 h	2 h
RDX	2.7 × 10^−05^	Acetonitrile [24]	0.16 at 313 nm [25]	7 h	1.8 d	
	7.2 × 10^−06^	Acetonitrile [26]		1.1 d	7 d	
	4.6 × 10^−05^	Water [25]		4 h	1.1 d	
metolachlor	1.6 × 10^−06^	Water [27]	9.6 × 10^−3^ at solar range [28], 3 × 10^−5^ at 313 nm [28]	5 d	1.5 y	9 d

Note:

a calculated using a reference spectrum of June 21 daily irradiance at 40 °N [18] and UV–vis spectra from the literature.

b The selected *φ* for half-life calculation is underlined.

c experiment conducted in spring at 45 °N, intensity approximately similar as the used reference solar spectrum [29].
